# Root Herbivores Drive Changes to Plant Primary Chemistry, but Root Loss Is Mitigated under Elevated Atmospheric CO_2_

**DOI:** 10.3389/fpls.2016.00837

**Published:** 2016-06-14

**Authors:** Scott W. McKenzie, Scott N. Johnson, T. Hefin Jones, Nick J. Ostle, Rosemary S. Hails, Adam J. Vanbergen

**Affiliations:** ^1^Centre for Ecology and Hydrology, EdinburghUK; ^2^The James Hutton Institute, DundeeUK; ^3^Centre for Ecology and Hydrology, WallingfordUK; ^4^School of Biosciences, Cardiff University, CardiffUK; ^5^Hawkesbury Institute for the Environment, University of Western Sydney, Sydney, NSWAustralia; ^6^Lancaster Environment Centre, Lancaster University, LancasterUK

**Keywords:** aphid, vine weevil, carbon, nitrogen, plant productivity, aboveground, belowground

## Abstract

Above- and belowground herbivory represents a major challenge to crop productivity and sustainable agriculture worldwide. How this threat from multiple herbivore pests will change under anthropogenic climate change, via altered trophic interactions and plant response traits, is key to understanding future crop resistance to herbivory. In this study, we hypothesized that atmospheric carbon enrichment would increase the amount (biomass) and quality (C:N ratio) of crop plant resources for above- and belowground herbivore species. In a controlled environment facility, we conducted a microcosm experiment using the large raspberry aphid (*Amphorophora idaei*), the root feeding larvae of the vine weevil (*Otiorhynchus sulcatus*), and the raspberry (*Rubus idaeus*) host-plant. There were four herbivore treatments (control, aphid only, weevil only and a combination of both herbivores) and an ambient (aCO_2_) or elevated (eCO_2_) CO_2_ treatment (390 versus 650 ± 50 μmol/mol) assigned to two raspberry cultivars (cv Glen Ample or Glen Clova) varying in resistance to aphid herbivory. Contrary to our predictions, eCO_2_ did not increase crop biomass or the C:N ratio of the plant tissues, nor affect herbivore abundance either directly or via the host-plant. Root herbivory reduced belowground crop biomass under aCO_2_ but not eCO_2_, suggesting that crops could tolerate attack in a CO_2_ enriched environment. Root herbivory also increased the C:N ratio in leaf tissue at eCO_2_, potentially due to decreased N uptake indicated by lower N concentrations found in the roots. Root herbivory greatly increased root C concentrations under both CO_2_ treatments. Our findings confirm that responses of crop biomass and biochemistry to climate change need examining within the context of herbivory, as biotic interactions appear as important as direct effects of eCO_2_ on crop productivity.

## Introduction

Root herbivory is very damaging to plants, especially when combined with multiple biotic and abiotic stresses (Zvereva and Kozlov, 2012) that can lead to substantial losses of crop yields (Villani and Wright, 1990; Blossey and Hunt-Joshi, 2003; Blackshaw and Kerry, 2008). Crop traits such as compensatory growth are key to crop survival and primary productivity in the face of herbivore pest pressure (Strauss and Agrawal, 1999; Watts et al., 2011; Huang et al., 2012; Robert et al., 2014). Plants, however, generally are less able to compensate for root herbivory compared to shoot herbivory (Johnson et al., 2016a). Moreover, even in simple agroecosystems insect herbivores occur as part of an above–belowground community (Megías and Müller, 2010; Soler et al., 2012). Consequently, the direct and indirect (mediated by host-plant plasticity) interactions among plants and herbivores occupying different guilds or niches, are key to understanding crop resistance and resilience to herbivory (Johnson et al., 2009; Huang et al., 2013; McKenzie et al., 2013; Hagenbucher et al., 2014).

Environmental stressors such as drought, elevated atmospheric CO_2_ (eCO_2_) and temperature can modify these trophic interactions (Johnson et al., 2011b; Stevnbak et al., 2012; Ryalls et al., 2013; Johnson et al., 2016a,b). Atmospheric CO_2_ concentrations are predicted to continue increasing during the 21st century and this is likely to affect plant productivity directly (Ainsworth and Long, 2005; Leakey et al., 2009; IPCC, 2013). For instance, greater accrual of plant biomass or altered biochemistry is one outcome of eCO_2_ (e.g., Hentley et al., 2014; Dáder et al., 2016). However, such effects may vary greatly due to intrinsic differences between plant species or the presence of other environmental stressors such as water stress or herbivory (Ainsworth and Long, 2005; Bader et al., 2009; Kohler et al., 2009; Johnson et al., 2011a; Johnson and Riegler, 2013). Changes to plant productivity has the potential to affect the performance of herbivores via changes in the quality (e.g., altered C and N content) of their plant food resource (DeLucia et al., 2012; Robinson et al., 2012). For example, in an eCO_2_ environment concentrations of N typically decrease by 17% in leaves and by 7% in roots (Robinson et al., 2012). This results in higher C:N ratios in plant tissues which generally reduces host plant quality for herbivores (Luo et al., 2006; Dáder et al., 2016), but this is a far from universal response. Many insect taxa respond idiosyncratically depending on species (e.g., aphids: Bezemer et al., 1999; Newman et al., 2003; Sun and Ge, 2011; Dáder et al., 2016; Ryalls and Harrington, 2016; Trębicki et al., 2016) or empirical information is so scarce for other groups (e.g., Staley and Johnson, 2008) that we cannot generalize either way. Moreover, while plant biomass or nutrient levels may alter in an eCO_2_ environment this may be moderated by the effects of herbivory. For instance, Johnson and Riegler (2013) showed concomitant increases in root herbivory in *Eucalyptus* seedlings, reversed several of the effects of elevated CO_2_ on plant growth and chemistry.

Herbivores shape plant primary productivity either by manipulating chemistry directly (e.g., aphid induced changes in source–sink relations; Crawley, 1989) or causing the plant to mobilize resources away from sites of attack (e.g., induced resource sequestration; Orians et al., 2011). Induced resource sequestration is thought to be a tolerance strategy to relocate resources temporarily away from the attacker (Kaplan et al., 2008; Schultz et al., 2013). This has traditionally focussed on plant attack aboveground, with photoassimilate transported to the roots for storage following shoot herbivory. Whether plants translocate primary compounds in the reverse direction in response to root herbivory has been subject to recent debate (Johnson et al., 2016a,b). Evidence is limited, but Robert et al. (2014) showed that maize plants infested with root herbivores allocated carbon to the stems as a prelude to root regrowth. Similarly, nitrogen reallocated from roots to shoots in knapweed (Newingham et al., 2007) and the stems in milkweed (Tao and Hunter, 2013) following root attack. It has been suggested, however, that root herbivores may manipulate their hosts to allocate primary metabolites belowground to improve host plant quality (Erb et al., 2013). Indeed, there is evidence that root herbivory causes increases in root carbon (Pierre et al., 2012; Robert et al., 2014) and blackcurrant (*Ribes nigrum*) plants attacked by root-feeding vine weevils had 72% lower concentrations of foliar phosphorus, with a concomitant increase of 56% in the roots (Johnson et al., 2013). In the present study, we term this ‘feeding-induced resource accumulation.’

It is clear that herbivores have the capacity to moderate plant primary chemistry and these impacts may vary at different CO_2_ concentrations. In this study we investigate how eCO_2_ influences plant (red raspberry *Rubus idaeus* L.) growth and primary chemistry when under attack from an aboveground (large raspberry aphid – *Amphorophora idaei* Börner) and belowground (vine weevil larvae – *Otiorhynchus sulcatus* F.) herbivore. Moreover, these two herbivores are thought to influence one another positively when sharing a host plant (McKenzie et al., 2013). In this study, we hypothesized that atmospheric carbon enrichment would alter the amount and quality of resources for herbivore species thus altering crop susceptibility to herbivory. Specifically we predicted that:

(i) eCO_2_ would cause an increase in plant biomass and the C:N ratio of above and belowground plant tissues,
(ii) the CO_2_ driven increase in host-plant biomass would result in greater herbivore abundance, above and belowground, but this may be negated by high C:N reducing host-plant quality
(iii) root herbivory will impede crop biomass gains under eCO_2_ and alter plant primary chemistry, via one or more mechanisms including impaired uptake of N, induced resource sequestration or feeding-induced resource accumulation.

## Materials and Methods

### Experimental Design

A microcosm experiment was carried out with 192 individual raspberry plants challenged with multifactorial combinations of herbivore, cultivar, and CO_2_ treatments. The experiment was performed in three runs (64 plants × 3 occasions) to avoid pseudoreplication and with CO_2_ treatments switched between different chambers per run to avoid any potential influence of chamber identity on the experiment. Each experimental run was of 10-weeks duration so the whole experiment spanned in total the period November 2011 – November 2012. Two cultivars (Glen Ample or Glen Clova), which varied in resistance to insect herbivory (Glen Clova was selectively bred for resistance to aphid herbivory), were exposed to an herbivore treatment comprising four levels: (i) herbivore-free control, (ii) aphid only, (iii) weevil only, and (iv) both herbivores present (12 plant replicates each). These herbivore × cultivar combinations were further challenged by exposure to either ambient (390 ± 50 μmol/mol) or elevated (650 ± 50 μmol/mol) atmospheric CO_2_ concentrations (*n* = 96), with the latter based on Climate Change (2007) predictions of atmospheric CO_2_ concentrations by 2100. Individual plant^TM^ replicates were assigned to randomized blocks within four controlled environment chambers (~4 m × 9 m) of the GroDome^TM^ climate change research facility at the Centre for Ecology and Hydrology (CEH), Wallingford, UK. A CO_2_ sensor (GMW22; Vaisala, Finland) in every chamber and was connected to a controller unit (AL2-24MR-D micro-controller, Mitsubishi, Japan). If CO_2_ levels fell below the treatment level (390 and 650 μmol/mol, respectively), CO_2_ gas (BOC, UK) was injected for 1 s, followed by a 30 s delay, repeating until the required atmospheric concentration was reached.

Individual plants were grown for 10-weeks from rootstock in the CO_2_ treatment chambers to which they were assigned. Photoperiod was maintained at 16:8 h (light:dark) with additional lighting provided by halide bulbs (400W) when photosynthetic active radiation (PAR) dropped below 400 μmol/s/m^2^, and a controlled daytime temperature of 18°C (±2°C) and minimum night temperature of 10°C (±2°C). Weevil eggs collected from cultures maintained at 18°C were added (20 per replicate) to the soil of appropriate replicates (weevil only and both herbivore treatment) in Week 4, with egg hatch occurring some 2 weeks later (Son and Lewis, 2005). Three adult large raspberry aphids were added to the upper-most unfurled leaf of the appropriate plants (aphid only and both herbivore treatment) in Week 8. The chronological sequence of weevil and aphid colonization of host-plants simulated in this experiment mimics the natural phenology of these organisms observed in the field (Moorhouse et al., 1992; McMenemy et al., 2009).

### Plant and Insect Sampling

After 10 weeks, aphid population sizes were determined by counts and removal of individuals. Vine weevil larvae were extracted from the soil for 24 h with Tullgren funnels and counted. Plants were carefully removed from the soil, roots washed and a random sample of leaves and roots was taken and snap-frozen in liquid nitrogen for analysis of plant primary chemistry. The remainder of the aboveground (stems, leaves) and belowground (root) plant biomass was then oven-dried (80°C for 24 h) and weighed (g). After being snap-frozen the roots and shoot samples were freeze dried for 24 h, then the tissue samples (≤5 mg) were ball-milled to a fine powder for subsequent C:N analysis. Chemical analysis of carbon and nitrogen concentrations of leaf and root tissue was undertaken at the Centre for Ecology and Hydrology (Lancaster), using an Exeter Analytical Elemental Analyser (EAI, Coventry, UK).

### Statistical Analysis

Co-linearity amongst parameters of plant biomass and biochemistry was initially assessed testes with Pearson correlation coefficients (proc CORR in SAS version 9.3). Subsequently, the response of plant biometrics (above- and belowground biochemistry and biomass) and herbivore abundance (aphid and weevil counts) to experimental treatments were analyzed with generalized linear mixed effects models (proc GLIMMIX). Categorical experimental treatments were: ‘herbivore’ (herbivore-free control, aphid only, weevil only, both herbivores), ‘Cultivar’ (Glen Ample or Glen Clova) and ‘CO_2_ regime’ (aCO_2_ or eCO_2_). For models of insect herbivore abundance, ‘herbivore treatment’ was replaced by continuous predictors: above- or belowground plant dry weight, % concentration of C, N, or C:N ratio of leaves or roots. Plant responses were modeled with Gaussian distribution and an identity link function, plant biomass was log transformed to meet the assumption that residuals were normally distributed with homogeneity of variance. Aphid and weevil counts were modeled with a Poisson distribution and a log link function.

Random effects were fitted to all models to account for different chambers used during the three experimental runs (chamber nested within run) and the randomized block design (block). Over-dispersion of count data in herbivore abundance models was accounted for with an observation-level parameter ‘plant replicate’ fitted as an additional random effect (Elston et al., 2001). The full model (experimental treatments and their pairwise interactions) was simplified through backward stepwise elimination of the least significant term (interactions before main effects) until a minimum adequate model was obtained. *F*-ratios and *p*-values reported are adjusted (SAS type III) for the other significant parameters retained in the final reduced model. Statistical significance of main effects are always reported, whereas two-way interactions are reported only where *P* < 0.05. Degrees of freedom were estimated using the Satterthwaite approximation (Littell et al., 1996). Least square means (with Bonferroni adjusted *p*-values) were plotted to show the effect of the significant explanatory variables conditional on other effects in the final models.

## Results

### Crop Biomass

Above- and belowground biomass were positively correlated (0.67; *p* < 0. 0001). In contrast to our prediction, eCO_2_ concentrations did not increase crop biomass overall, either aboveground (*F*_1,4_ = 1.78, *p* = 0.2544) or belowground (*F*_1,4_ = 3.54, *p* = 0.1345). There was, however, an interaction between CO_2_ treatment and crop cultivar (*F*_1,175_ = 4.52, *p* = 0.0349), explained by cv. Glen Ample accruing greater aboveground biomass than cv. Glen Clova at eCO_2_ levels (Bonferroni adjusted *p* = 0.0252).

**FIGURE 1:**
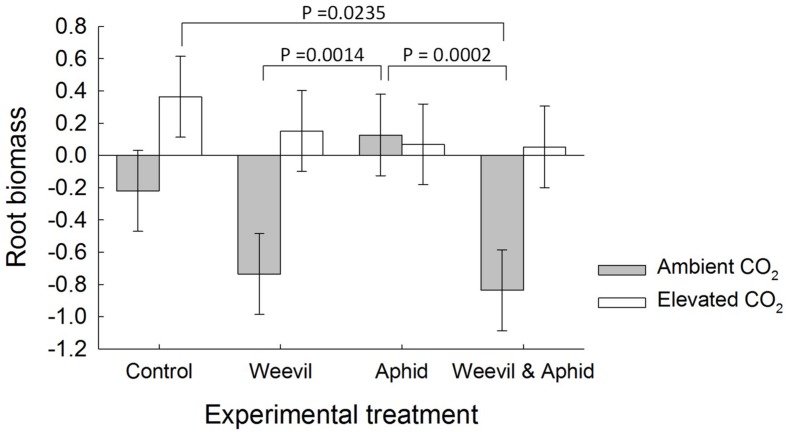
**The effect on raspberry root biomass of CO_2_ treatment (dark bars = ambient 390 ± 50 μmol/mol; light bars = elevated 650 ± 50 μmol/mol) and herbivore treatments (herbivore-free control, root-feeding weevil only, foliar-feeding aphid only, both herbivores).** Data are least square means ± SE derived from final GLMM accounting for variation due to other treatments. Difference among treatments following Bonferroni adjustment for multiple comparisons indicated with solid lines (*p* < 0.05).

Although there was no indication of any effect of herbivore treatment on aboveground biomass (*F*_3,173_ = 0.44 *p* = 0.7275), root herbivory consistently reduced root biomass with treatments where weevil larvae were present (weevil only, both herbivore species) yielding significantly less root biomass than treatments without weevils (control and aphid only; *F*_3,172_ = 5.88, *p* = 0.0008, **Figure 1**). Root biomass was also affected by the significant interaction between the herbivore and CO_2_ treatments (*F*_3,172_ = 4.66, *p* = 0.0037, **Figure 1**). While under aCO_2_ conditions root biomass was significantly reduced by treatments including root-feeding weevils (weevils only and both herbivore species), this effect dissipated under eCO_2_ (**Figure 1**), suggesting a mitigation of herbivory on roots.

The identity of the crop cultivar also had an influence on above- and belowground crop biomass. Aboveground biomass was greatest in the cultivar (Glen Clova) selectively bred to be most resistant to aphid herbivory (Glen Clova LS mean = -0.32 ± 0.17; Glen Ample LS mean = -0.19 ± 0.17; *F*_1,175_ = 3.93, *p* = 0.0349). Whereas, belowground biomass was significantly greater in the cultivar (Glen Ample) that was less resistant to aphid herbivory (Glen Clova LS mean = -0.03 ± 0.16; Glen Ample LS mean = -0.23 ± 0.16; *F*_1,171_ = 4.17, *p* = 0.0427).

### Crop Biochemistry

Correlation analysis revealed the intimately connected balance of C and N within the crop plant and these relationships are shown in Supplementary Material (Appendix S1).

As with aboveground crop biomass, and contrary to prediction, the experimental eCO_2_ treatment had little overall impact on plant tissue biochemistry. There was only a slight increase in percent leaf C (LS mean: ambient = 42.27, elevated = 42.98 ± 0.1658; *F*_1,4_ = 9.24, *p* = 0.0388), with little overall effect on leaf N (*F*_1,4_ = 6.26, *p* = 0.0672) and hence the C:N ratio of leaves (*F*_1,4_ = 6.47, *p* = 0.0666). The CO_2_ treatment had no effect on the percent C (*F*_1,4_ = 0.00, *p* = 0.9968), percent N (*F*_1,4_ = 0.50, *p* = 0.5207) or the C:N ratio (*F*_1,4_ = 0.59, *p* = 0.4909) of roots.

**FIGURE 2:**
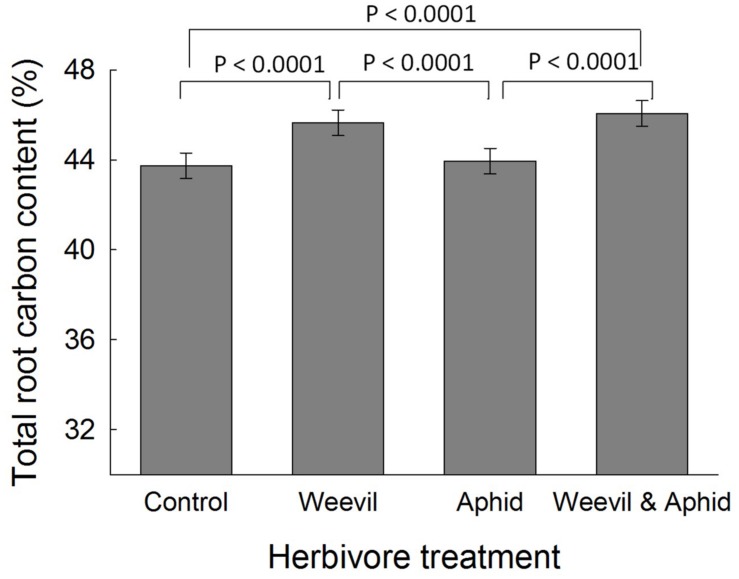
**The effect of herbivore treatment (herbivore-free control, root-feeding weevil only, foliar-feeding aphid only, both herbivores) on the carbon content (%) of raspberry roots.** Data are least square means ± SE derived from final GLMM accounting for variation due to other treatments. Difference among treatments following Bonferroni adjustment for multiple comparisons indicated with solid lines (*p* < 0.05).

**FIGURE 3:**
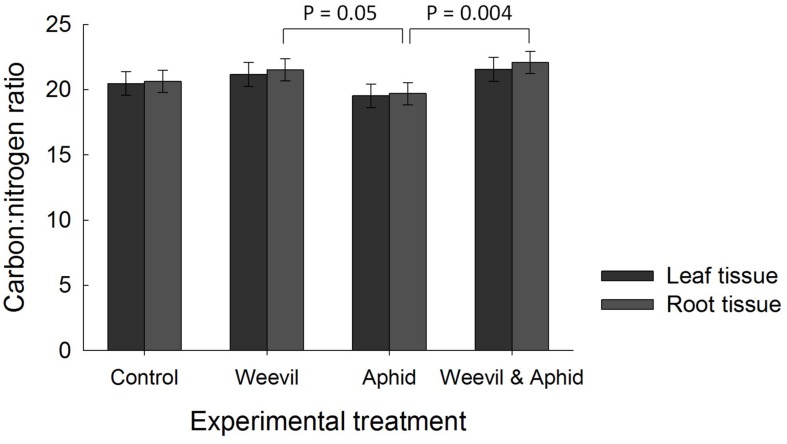
**The effect of herbivore treatment (herbivore-free control, root-feeding weevil only, foliar-feeding aphid only, both herbivores) on the ratio of carbon to nitrogen (C:N) in raspberry leaf (dark bars) and root (light bars) tissues.** Data are least square means ± SE derived from final GLMM accounting for variation due to other treatments. Difference among treatments following Bonferroni adjustment for multiple comparisons indicated with solid lines (*p* < 0.05).

There was no evidence that the herbivore treatment affected the overall percent content of C (*F*_3,177_ = 0.98, *p* = 0.4019) or N (*F*_3,174_ = 1.82, *p* = 0.1452) or the C:N ratio (*F*_3,169_ = 2.00, *p* = 0.1158, **Figure 3**) of leaf tissues. While root herbivory did not significantly affect belowground N content (*F*_3,174_ = 2.24, *p* = 0.0851), it did greatly increase the C content of root tissues relative to control and aphid treatments (*F*_3,171_ = 30.99, *p* < 0.0001, **Figure 2**). This herbivore effect was reflected in a higher C:N ratio (*F*_3,174_ = 4.68, *p* = 0.0036) in roots where belowground herbivory was present, relative to the aphid-only herbivore treatment (**Figure 3**).

**FIGURE 4:**
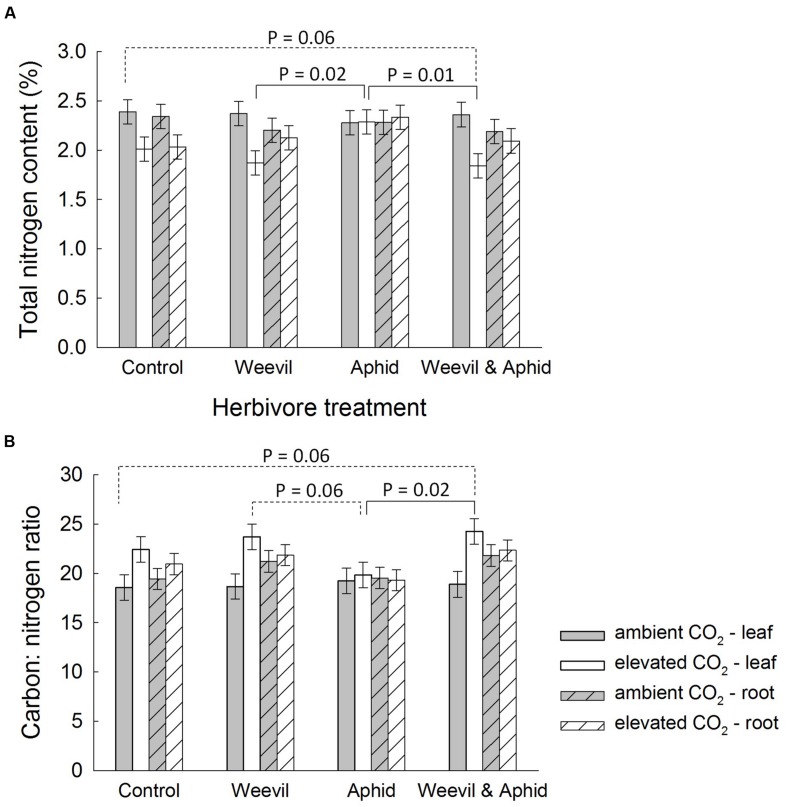
**The effect on raspberry **(A)** carbon to nitrogen (C:N) ratio and **(B)** nitrogen content (%) of the interaction between CO_2_ (dark bars = ambient 390 ± 50 μmol/mol; light bars = elevated 650 ± 50 μmol/mol) and herbivore treatments (herbivore-free control, root-feeding weevil only, foliar-feeding aphid only, both herbivores).** Data are least square means ± SE derived from final GLMM accounting for variation due to other treatments. Difference among treatments following Bonferroni adjustment for multiple comparisons indicated with dashed (marginally non-significant) or solid lines (*p* < 0.05).

Furthermore, similar to the effect of root herbivory on belowground biomass (see above), the interaction between the herbivore and CO_2_ treatments affected percentage N (*F*_3,174_ = 4.02, *p* = 0.0085) and C:N ratio (*F*_3,169_ = 3.01, *p* = 0.0319) of leaves. At aCO_2_ conditions, the leaf N content (**Figure 4A**) and C:N ratio (**Figure 4B**) was unaffected by root-feeding weevils or foliar-feeding aphids. Under eCO_2_ conditions, however, root-feeding weevils generally decreased N content (**Figure 4A**) and hence increased the aboveground C:N ratio (**Figure 4B**).

Crop cultivar affected the C content of above- and belowground tissues. Leaf C content was generally greater in cultivar Glen Clova (LS mean = 42.89 ± 0.13) than Gl. Ample (LS mean = 42.36 ± 0.13; *F*_1,180_ = 15.83, *p* = 0.0001). Root C content was similarly higher in Glen Clova (LS mean = 445.38 ± 0.55) than Glen Ample (LS mean = 44.33 ± 0.55; *F*_1,169_ = 24.62, *p* < 0.0001). The interaction between the CO_2_ treatment and cultivar also affected crop biochemistry, with the greatest effects in aboveground tissues (**Table 1**). The C content of Glen Clova leaves was increased significantly by exposure to an eCO_2_ environment, whereas Glen Ample was largely unaffected (**Table 1**). While the impact on root C content was generally lower, there was a significant difference in the response of the cultivars to eCO_2_ with Glen Clova allocating more C to roots (**Table 1**). Similarly, leaf N content was lowered by CO_2_ treatment in both cultivars, but was most pronounced in the Glen Clova cultivar, while root N was largely unaffected by this interaction (**Table 1**). These shifts in the crop biochemical balance translated into a highly significant increase in the aboveground C:N ratio following exposure to an eCO_2_ environment, largely driven by the cultivar most resistant to herbivory (Glen Clova; **Table 1**).

**Table 1:** The effect on crop primary biochemistry of the interaction between crop cultivar and experimental CO_2_ treatment.

Cultivar	Glen Clova		Glen Ample		*F*(df)	*P*
CO_2_ regime	390 μmol/mol	650 μmol/mol	390 μmol/mol	650 μmol/mol		
**Leaf**						
Nitrogen (%)	2.49 ± 0.11	1.96 ± 0.11	2.21 ± 0.11	2.04 ± 0.11	8.38 (1, 174)	0.0043
Carbon (%)	42.34 ± 0.19	43.44 ± 0.19	42.20 ± 0.19	42.52 ± 0.19	8.55 (1, 180)	0.0039
C:N	17.75 ± 1.13	23.34 ± 1.13	19.91 ± 1.13	21.74 ± 1.13	8.90 (1, 169)	0.0033
**Root**						
Nitrogen (%)	2.34 ± 0.11	2.14 ± 0.11	2.17 ± 0.11	2.15 ± 0.11	3.78 (1, 173)	0.0535
Carbon (%)	45.16 ± 0.77	45.59 ± 0.77	44.54 ± 0.77	44.11 ± 0.77	4.20 (1, 169)	0.0420
C:N	19.78 ± 1.09	21.96 ± 1.09	21.08 ± 1.09	21.11 ± 1.09	4.99 (1,173)	0.0268

*Data are least-square means and F & P values derived from final GLMM for each crop parameter.*

### Insect Herbivore Responses

**FIGURE 5:**
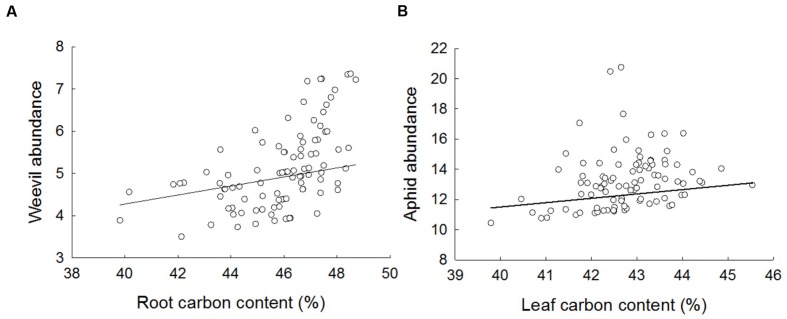
**The effect on the abundance of **(A)** weevils and **(B)** aphids of percent root and leaf carbon, respectively.** Data are partial residual plots on the linear predictor scale and fitted lines are from final GLMM slopes accounting for variation due to other treatments and random effects.

Aphid abundance was weakly but positively related to leaf C content (**Figure 5**; *F*_1,77_ = 4.47, *p* = 0.0378). There was no statistically significant evidence that aphid abundance was related to either aboveground crop biomass (*F*_1,69_ = 2.77, *p* = 0.0770), leaf N content (*F*_1,81_ = 3.44, *p* = 0.0674) or the leaf C:N ratio (*F*_1,74_ = 1.16, *p* = 0.2860). Weevil abundance was positively related to root C content (**Figure 5**; *F*_1,76_ = 5.56, *p* = 0.0210), but not root N (*F*_1,83_ = 0.41, *p* = 0.5253) or belowground biomass (*F*_1,71_ = 1.80, *p* = 0.1838) or the root C:N ratio (*F*_1,80_ = 0.160, *p* = 6862).

Despite bred resistance to aphid herbivory (cv. Glen Clova), there was no significant differences in insect herbivore abundance between the cultivars (aphid: *F*_1,69_ = 0.48, *p* = 0.4894; weevil: *F*_1,68_ = 0.63 *p* = 0.4311) nor was there any direct effect of the CO_2_ treatments on herbivore abundance (aphid: *F*_1,4_ = 3.58, *p* = 0.4957; weevil: *F*_1,4_ = 0.55, *p* = 0.4996).

There was no evidence that the abundance of each herbivore was influenced by the abundance of the other species (weevil: *F*_1,37_ = 3.01, *p* = 0.0911; aphid: *F*_1,26_ = 2.44, *p* = 0.1305), and hence no indication of a positive or negative plant-mediated herbivore interaction in this study.

## Discussion

Contrary to our first prediction, eCO_2_ did not directly increase crop biomass or the C:N ratio of the plant tissues. Enhanced growth rates in response to eCO_2_ are common (Hentley et al., 2014; Dáder et al., 2016), especially in C3 plant species that at current CO_2_ concentrations operate below the maximum capacity of the carboxylating plant enzyme Rubisco (Ainsworth and Long, 2005; Leakey et al., 2009). These gains in biomass, however, range between 0 and 20% depending on plant species or functional type, for instance tree species typically accrue greater biomass than cereal crops or many wild herbaceous species (Ainsworth and Long, 2005; Ainsworth and Rogers, 2007; DeLucia et al., 2012). Furthermore, plant growth can even decrease in response to eCO_2_ according to the presence of other environmental stressors, such as water availability (Bader et al., 2009; Kohler et al., 2009). Herbivores can also offset any plant biomass gain due to eCO_2_ by compensating for lower host-plant quality (e.g., reduced N content) by increasing or maintaining feeding rates through behavioral or physiological plasticity (Barbehenn et al., 2004; Johnson et al., 2011a).

Aphid and weevil abundance were independent of atmospheric CO_2_ concentrations, therefore there was also no evidence to support our second prediction that eCO_2_ would increase insect herbivore abundance. This finding fits among the many examples of aphids showing positive, negative or neutral responses to CO_2_ treatments (Bezemer et al., 1999; Newman et al., 2003; Sun and Ge, 2011; Dáder et al., 2016; Trębicki et al., 2016). Elsewhere, the nitrogen status (e.g., C:N ratio) of plant tissues has been shown to be intimately related to life-history or population performance of other aphid species under eCO_2_ (e.g., *Myzus persicae* Sulzer – Dáder et al., 2016; *Rhopalosiphum padi L.* – Trębicki et al., 2016). For instance, eCO_2_ decreased the foliar N content, but not the C content, in pepper plants (*Capsicum annum* L.) leading to longer individual development and lower fecundity of *Myzus persicae* due to an unfavorable nutritional quality of the host-plant (Dáder et al., 2016). In this experiment, the comparatively weak effects of eCO_2_ on the nitrogen balance in these raspberry cultivars offer a potential explanation for the lack of an effect on the aphid or weevil herbivore. Although unquantified here, this lack of a profound eCO_2_ effect on the C–N balance implies it was unlikely to have modified the herbivore nutrients (e.g., essential amino acids) or the physical (e.g., cuticular waxes) or secondary (i.e., salicylic acid signaling pathway) defenses governing crop-herbivore interactions (Sun and Ge, 2011).

To understand better crop performance in eCO_2_ environments more work is clearly needed to unravel the interplay between, biochemical state, insect nutrition and performance in different crop varieties. In agreement with our study, Hentley et al. (2014) showed *A. idaei* did not respond to eCO_2_ when reared on these same raspberry cultivars (Glen Ample and Glen Clova) in the absence of the competing belowground herbivore. Similarly, Martin and Johnson (2011) also reported that *A. idaei* was unaffected by eCO_2_ on two other raspberry cultivars (Glen Rosa and Malling Jewel). However, aphid performance improved under eCO_2_ on other raspberry cultivars (Glen Lyon in Martin and Johnson, 2011; cv. Octavia – Hentley et al., 2014). These different outcomes among experiments and cultivars may point to the pre-dominance of the host-plant and insect identity over climate effects for herbivore performance, or just simply to experimental artifacts. Nonetheless, further experimental information on the role of different cultivars in shaping herbivory under climate change should continue to be an important avenue of research.

In terms of insect interactions, this experiment did not find evidence for the previously observed reciprocal feeding facilitation between these two spatially separated herbivores at aCO_2_ (McKenzie et al., 2013). Different crop growing conditions, use of different climate controlled facilities, and the fact that the current experiment was performed over a longer time-period (three 10-week runs over a calendar year vs. single run of 10 weeks) could explain this difference between these two studies.

Root herbivory affected root biomass and the C:N ratio of above- and belowground crop tissues and this was modified by the level of atmospheric CO_2_ that the crop experienced. In accord with our third prediction, root herbivory reduced belowground biomass significantly under aCO_2_ conditions, however, this impact dissipated under eCO_2_. This suggests a mitigation of herbivory on roots, potentially via impacts on herbivore performance at the individual or population level in an enriched CO_2_ atmosphere (Johnson et al., 2011a).

The most likely mechanism explaining the nullification of root herbivory is that increased concentrations of atmospheric carbon enable enhanced compensatory root re-growth, therefore lessening the net root loss. The net effect of the combination of root herbivory and eCO_2_ was similar to that found by Johnson and Riegler (2013), where the same combination produced root biomass at levels similar to those at aCO_2_ concentrations in the absence of herbivory. A notable difference is that Johnson and Riegler (2013) showed eCO_2_ to increase root biomass, which was subsequently reduced by herbivory; whereas here loss of biomass by root-herbivory under aCO_2_ conditions was mitigated by increased root production at eCO_2_. The net effect, however, remains the same with the abiotic and biotic pressures balancing one another.

Mirroring the change in crop biomass, the leaf C:N ratio was increased by root herbivory at eCO_2_, but not aCO_2_ conditions. This finding is consistent with our third prediction that root herbivores would cause changes in primary chemistry. We suggest that damage to roots from herbivory would restrict the uptake of nitrogen from the soil, as evidenced by the lower N concentrations in roots, and this likely shifted the C:N ratio in leaves (Zvereva and Kozlov, 2012). We found no support for induced resource sequestration (i.e., movement of C or N to the shoots) as a result of root herbivory, since foliar concentrations were not affected by either herbivore. On the contrary, we found evidence that root herbivores increased C concentrations in the roots. This may reflect ‘feeding-induced resource accumulation’ either because the herbivore is manipulating the plant for its own benefit, or the plant is mobilizing resources for root regrowth.

This study emphasizes the importance of understanding crop biomass and biochemical responses to climate change in the context of herbivory. In this system, biotic interactions appear as important as direct effects of climate change on crop productivity. Experimental work should continue to test how increasing the trophic complexity of the crop system affects species interactions and crop performance in a carbon-enriched world (Soler et al., 2012; Hentley et al., 2014; Dáder et al., 2016; Trębicki et al., 2016).

## Author Contributions

SM helped design and run the experiment, carried out measurements and contributed to analysis and paper preparation. THJ and RH helped conceive and design the experiment and contributed to the writing of the paper. AV helped conceive, design the experiment, analyzed the data, and prepared the paper. SJ helped conceive and design the experiment and prepared the paper. NO conceived and oversaw biochemical analysis of the experiment and contributed to the writing of the paper.

## Conflict of Interest Statement

The authors declare that the research was conducted in the absence of any commercial or financial relationships that could be construed as a potential conflict of interest.
